# Optimized bioluminescence analysis of adenosine triphosphate (ATP) released by platelets and its application in the high throughput screening of platelet inhibitors

**DOI:** 10.1371/journal.pone.0223096

**Published:** 2019-10-10

**Authors:** Lili Wang, Yunqian Li, Ran Guo, Shanshan Li, Anqi Chang, Zhixiang Zhu, Pengfei Tu

**Affiliations:** Modern Research Center for Traditional Chinese Medicine, School of Chinese Materia Medica, Beijing University of Chinese Medicine, Beijing, China; Broad Institute, UNITED STATES

## Abstract

Activated platelets release adenosine trisphosphate (ATP) and bioluminescence analysis of ATP release is usually used to monitor activation of platelets induced by various stimulants. However, bioluminescence analysis of ATP possesses poor linearity, the signal is quickly attenuated, and the accuracy of ATP release from platelets is hard to determine accurately enough to be used in a high throughput screening of platelet inhibitors. The present study was designed to optimize bioluminescence analysis of ATP released by platelets and expand its application in high throughput screening of platelet inhibitors. The results showed that accuracy of ATP analysis was significantly improved by adding coenzyme A (CoA) and signal attenuation of ATP analysis was greatly postponed by adding bovine serum albumin (BSA) both in Hank’s balanced salt solution (HBSS) and Tyrode’s buffer. Furthermore, ATP release of activated platelets and inhibitory effects of Ly294002 and Staurosporine on platelet activation were accurately determined by our optimized bioluminescence analysis of ATP. Thus, we have successfully constructed an optimized bioluminescence analysis of ATP which can be used in high throughput screening of platelet inhibitors.

## Introduction

Platelets are a component of blood and play important roles in hemostasis. In platelets, there are two different types of releasable granules which are named as alpha granules and dense granules. Alpha granules contain various presynthesized thrombogenic proteins, such as von Willebrand factor (vWF), fibronectin, and P-selectin. Dense granules contain small molecules, especially adenosine diphosphate (ADP), adenosine triphosphate (ATP) and serotonin [1]. After circulating platelets are exposed to collagen-rich subendothelium at the site of vascular injury or thrombin, these granules are secreted, which is essential for the recruitment and activation of further platelets, thereby promoting platelet aggregation, thrombus formation, and wound repair [2, 3]. On the other hand, platelet activation in pathologic situations can lead to thrombosis, causing myocardial infarction or stroke. In order to develop novel inhibitors of platelet activation for the prevention and treatment of thrombotic diseases, it is necessary to analyze platelet activation accurately and conveniently.

As quantitative determination of ATP based on bioluminescence reaction catalyzed by firefly luciferase is a superior method with high sensitivity and specificity, platelet activation can be detected through analyzing ATP release of activated platelets [4]. Therefore, bioluminescence analysis of ATP can also be used to screen platelet inhibitors [5–7]. However, there are still some defects in the quantitative analysis of ATP released by platelets, including poor accuracy and excessive attenuation of bioluminescence.

Luciferase catalyzes the oxidation of D-luciferin (LH_2_) with molecular oxygen in the presence of ATP and Mg^2+^ with emission of yellow-green light in a highly efficient process. This reaction involves the formation of luciferyl adenylate intermediate (LH_2_-AMP) with the release of pyrophosphate. LH_2_-AMP not only can be oxidized into oxyluciferin accompanied by release of AMP, carbon dioxide, and light emission, but also be oxidized into dehydroluciferyl adenylate (L-AMP) which is a side product (about 20% of the reacted LH_2_) of the bioluminescence reaction. Undesirably, L-AMP has been identified as a powerful luciferase inhibitor with an IC50 of about 6 nM and significantly impacts bioluminescence reaction rate of ATP analysis catalyzed by luciferase, especially at high concentration of ATP [8–10]. The inhibitory effects of L-AMP on bioluminescence reaction result in unsatisfactory linearity and accuracy of ATP analysis. Furthermore, quick consumption of ATP in the bioluminescence reaction leads to excessive signal attenuation. These defects in the bioluminescence analysis of ATP seriously restricted its application in the high throughput screening of platelet inhibitors.

In the present study, we investigated the effects of coenzyme A (CoA) on linearity improvement and the effects of bovine serum albumin (BSA) on signal attenuation in bioluminescence analysis of ATP. Subsequently, we explored applicability of optimized bioluminescence analysis of ATP in platelet activation assay and high throughput screening of platelet inhibitors.

## Materials and methods

### Reagents

A stock solution of luciferase (product number: L9420), 1, 4-dithiothreitol (DTT), adenosine 5’-triphosphate disodium salt (ATP), coenzyme A sodium salt (CoA), BSA, ovalbumin (OVA), luminol sodium salt, 4-(imidazol-1-yl)phenol (4-IMP), hydrogen peroxide solution (H_2_O_2_), peroxidase from horseradish (HRP) and thrombin were purchased from Sigma-Aldrich (St. Louis, MO, USA). D-luciferin sodium salt was purchased from J&K Scientific (Beijing, China). Collagen was purchased from Hyphen Biomed (Neuville-sur-Oise, France). Hank’s balanced salt solution (HBSS) was obtained from ThermoFisher Scientific (Waltham, MA, USA). Ly294002 (phosphoinositite 3 kinase (PI3K) inhibitor) and Staurosporine (protein kinase C (PKC) inhibitor) were purchased from Abmole Bioscience (Houston, TX, USA). Prostaglandin E1 (PGE1) and other commonly used chemical reagents were purchased from Aladdin Industrial (Shanghai, China).

### Animals

Twelve 3-month-old Male Sprague Dawley rats (body weight, 220 ± 10g) were purchased from SPF Biotechnology (Beijing, China). The rats were maintained at a constant temperature and humidity with a 12 h light/dark cycle, and free access to food and water. All protocols conformed to guidelines in Beijing University of Chinese Medicine (Beijing, China) and the study was approved by the Ethical Committee on Animal Research (2018-03-4328) in Beijing University of Chinese Medicine.

### Preparation of washed platelets

Blood was withdrawn from abdominal aorta of anesthetized rat into evacuated tube for blood specimen collection containing 3.8% sodium citrate as anticoagulant. Platelet-rich plasma (PRP) and washed platelets were prepared as described previously [1–3]. In brief, immediately after blood collection, rat’s blood was centrifuged at 200 g for 10 minutes at room temperature and the PRP was carefully separated. Platelets were separated from PRP by centrifugation at 1000 g for 10 minutes and washed with CGS buffer (1.7 mM citric acid, 18.3 mM trisodium citrate, 10 mM glucose, 120 mM sodium chloride, PH 6.5) in the presence of PGE1 (2 μM) and resuspended in buffers of subsequent experiments.

### Bioluminescence analysis of ATP by adding CoA

All the enzyme reactions were performed in white 96-well plates at ambient temperature (24–27°C). Firstly, a stock solution of ATP (10 mM) was serially diluted into 1 to 0.031 μM with buffer A (25 mM Tricine-HCl, 5 mM MgSO_4_, 0.1 mM EDTA, pH 7.8) and added into 96-well plate (100 μl). Then enzyme work solution (2 ×) containing DTT (2 mM), luciferin (1 mM) and luciferase (2.5 μg/ml) was also prepared by buffer A, and added into 96-well plate (100 μl) to initiate bioluminescence reaction. Unless otherwise stated, the plates of bioluminescence analysis were shaken for 10 s after all reagents were added. Relative light unit (RLU) was analyzed on EnSpire Multimode Plate Reader (PerkinElmer, Waltham, MA, USA) at different time points. Secondly, when the concentration of ATP in the enzyme reaction mixture was fixed at 1 μM, a series of concentrations of CoA were added in reaction system and the effects of CoA on bioluminescence assay were analyzed. At last, determination of standard curve of ATP was performed again in the presence of optimized concentration of CoA (15 μM).

### Bioluminescence analysis of ATP in HBSS and Tyrode’s buffer

ATP was serially diluted into 1 to 0.031 μM with HBSS (138 mM NaCl, 5.33 mM KCl, 0.34 mM Na_2_HPO_4_, 0.44 mM KH_2_PO_4_, 4.17 mM NaHCO_3_, 5.56 mM glucose, 0.41 mM MgSO_4_, 0.49 mM MgCl_2_ and 1.26 mM CaCl_2_, pH 7.3) or modified Tyrode’s buffer (137 mM NaCl, 2.9 mM KCl, 0.34 mM Na_2_HPO_4_, 12 mM NaHCO_3_, 5 mM HEPES, 5 mM glucose, 1 mM MgCl_2_ and 1 mM CaCl_2_, pH 7.3) and added into 96-well plate (100 μl). In the presence or absence of CoA (30 μM), enzyme work solution (2 ×) containing DTT (2 mM), luciferin (1 mM) and luciferase (2.5 μg/ml) was also prepared with HBSS or Tyrode’s buffer, and added into 96-well plate (100 μl) to initiate bioluminescence reaction, then RLU was analyzed at different time points.

### Bioluminescence analysis of ATP by adding BSA in HBSS and Tyrode’s buffer

ATP was diluted into 1 μM with HBSS or Tyrode’s buffer and added into 96-well plate (100 μl). A series of concentrations of BSA were added in reaction system (50 μl). Ultimately, enzyme work solution (4 ×) containing DTT (4 mM), luciferin (2 mM), luciferase (5 μg/ml) and CoA (60 μM) was added into 96-well plate (50 μl) to initiate bioluminescence reaction and RLU was analyzed at different time points. Subsequently, a series of concentrations of ATP were added into 96-well plate (100 μl). In the presence or absence of BSA (0.05%), the enzyme work solution (2 ×) containing DTT (2 mM), luciferin (1 mM), luciferase (2.5 μg/ml) and CoA (30 μM) was added into 96-well plate (100 μl) to initiate bioluminescence reaction and RLU was analyzed at different time points.

### Chemiluminescence analysis of HRP by adding BSA and bioluminescence analysis of ATP by adding OVA

In the presence of absence of 0.05% BSA, enhanced chemiluminescence reaction of luminol and H_2_O_2_ was initiated by adding HRP with a final concentration of 0.001 U/ml into 200 μl reaction solution (0.1 M Tris-HCl, 1.25 mM luminol, 3 mM H_2_O_2_, 0.5 mM 4-IMP, pH 8.6). RLU was analyzed at different time points. In addition, bioluminescence analysis of ATP (1 μM) was performed in 200 μl reaction system (1 mM DTT, 1 mM luciferin, 1.25 μg/ml luciferase and 15 μM CoA) with or without 0.05% OVA at different time points.

### Method validation of ATP analysis in the presence of platelets and bioluminescence analysis of ATP released by activated platelets

Washed platelets were suspended in HBSS or Tyrode’s buffer (2 × 10^7^ platelets/ml) and seeded in white 96-well plate (100 μl). In the presence or absence of CoA (15μM), standard curves of ATP analyses in HBSS or Tyrode’s buffer containing platelets were determined again. In addition, in the presence or absence of BSA (0.05%), signal attenuations of ATP analyses in HBSS or Tyrode’s buffer containing platelets were evaluated. Recovery rates of ATP were investigated by adding standard ATP (0.1μM or 1 μM) into HBSS or Tyrode’s buffer containing platelets and performing quantitative analysis of ATP. At last, the platelet suspension was added into 96-well plate (100 μl) or 384-well plate (20 μl). Subsequently, the platelets were stimulated with thrombin (0.1 U/ml) or collagen (20 μg/ml) at 37°C for 20 min. Bioluminescence analysis of ATP released by activated platelets was initiated by adding enzyme work solution containing CoA and BSA into reaction system and RLU was determined at 5 min after the start of the reaction.

### Screening of platelet inhibitors with optimized bioluminescence analysis of released ATP

Washed platelets were resuspended in HBSS or Tyrode’s buffer (2 × 10^7^ platelets/ml) and seeded in white 96-well plate (100 μl). The platelets were pretreated with or without 25 μl compounds for 5 min and stimulated with 25 μl thrombin (0.6 U/ml) or collagen (120 μg/ml) at 37°C for 20 min. Bioluminescence analysis of ATP released by activated platelets was initiated by adding enzyme work solution (4 ×, 50 μl) into 96-well plate and RLU was determined at 5 min after the start of the reaction.

### Statistical analysis

The results were expressed as mean ± standard deviation (SD) of triplicates from one representative experiment or three independent experiments as described. Z’ factor was calculated with the formula: Z’ factor = 1 - (3 × (SD_agonist_ + SD_vehicle_) / (Mean_agonist_—Mean_vehicle_). For each condition, Z’ factor was acquired from 96 samples in 96-well plate or from 384 samples in 384-well plate. Half were stimulated platelets and half were non-stimulated platelets. Statistical analysis was performed using GraphPad Prism 5. Unpaired student’s t test or one-way ANOVA followed by Dunnett’s test was used to evaluate differences between experimental groups and P value less than 0.05 was considered to be statistically significant.

## Results

### Effects of CoA on the bioluminescence analysis of ATP in buffer A

**Fig 1:**
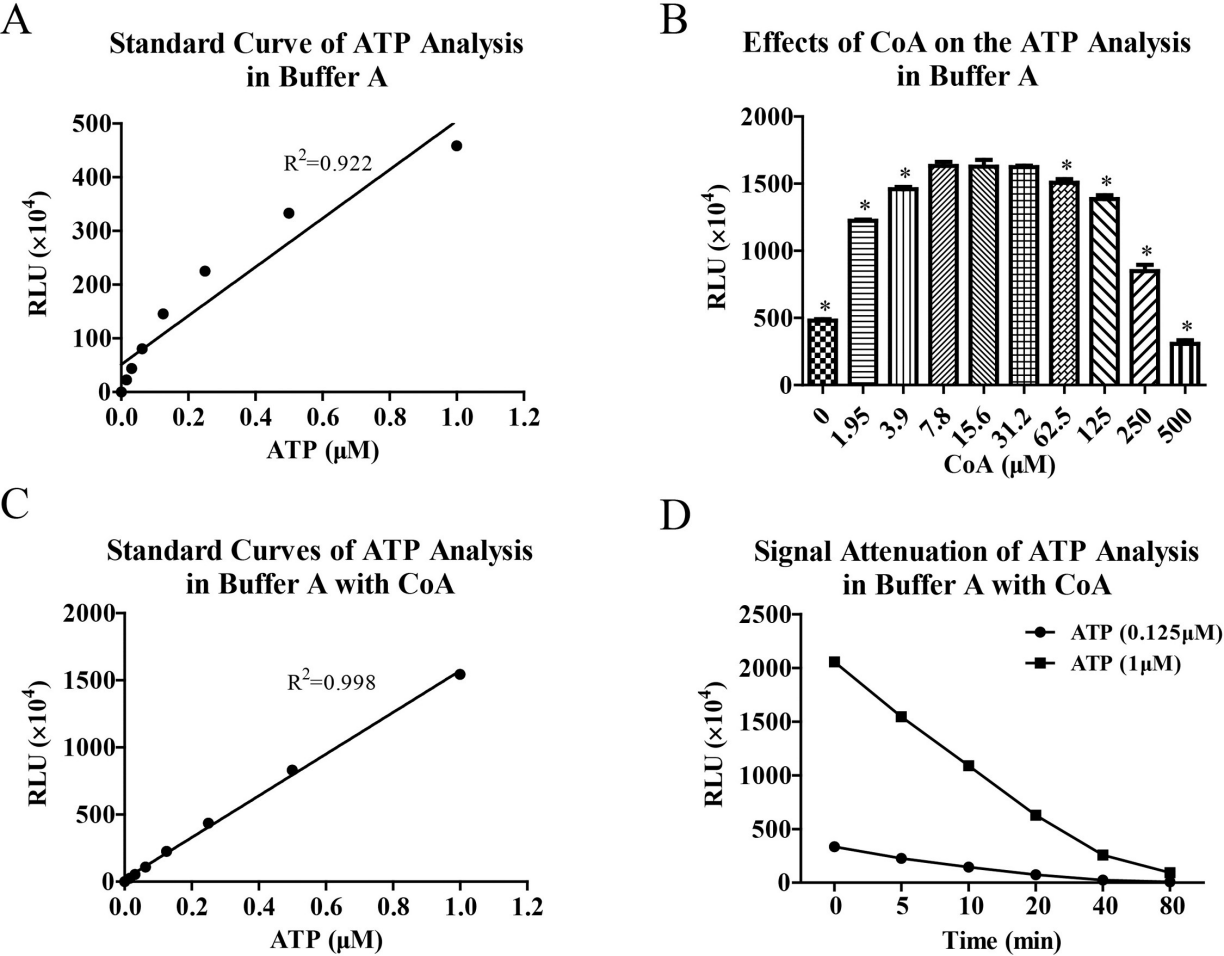
**CoA improved linear range of bioluminescence analysis of ATP in buffer A.** Bioluminescence analysis of ATP was performed in buffer A and standard curve was made (A). The effects of CoA on the signal intensity of ATP analysis were evaluated (B). Then standard curve analysis of ATP (C) was made again in the presence of CoA at optimized concentration (15 μM) and signal attenuation of ATP analysis (D) was also estimated by determining relative light unit (RLU) at different time points. Data are representative of three independent experiments. * *P* < 0.05 *vs* CoA (15.6 μM).

In the bioluminescence analysis of ATP, we first performed standard curve assay in buffer A. As shown in Fig 1A, standard curve of ATP analysis exhibited unsatisfactory linearity and R^2^ of linear regression analysis was only 0.922. Obviously, RLU values were not proportional to the concentrations of ATP when ATP was at high concentrations. Because CoA can block inhibitory effects of enzyme reaction product on luciferase activity, we attempted to improve linearity of ATP analysis by adding CoA in the bioluminescence reaction system. The results (Fig 1B) showed that RLU values of ATP analysis were significantly affected by CoA and CoA in the concentration range of 7.8 to 31.2 μM enhanced RLU values of ATP analysis to the maximum extent. Thus, we carried out standard curve analysis of ATP again by adding 15 μM CoA in the reaction system. As shown in Fig 1C, the standard curve of ATP analysis was greatly improved and R^2^ was 0.998. However, the signal attenuation of ATP analysis in buffer A was quick even in the presence of CoA and attenuation rate reached more than 60% in 20min and 90% in 80 min (Fig 1D).

### Bioluminescence analyses of ATP in HBSS and Tyrode’s buffer

**Fig 2:**
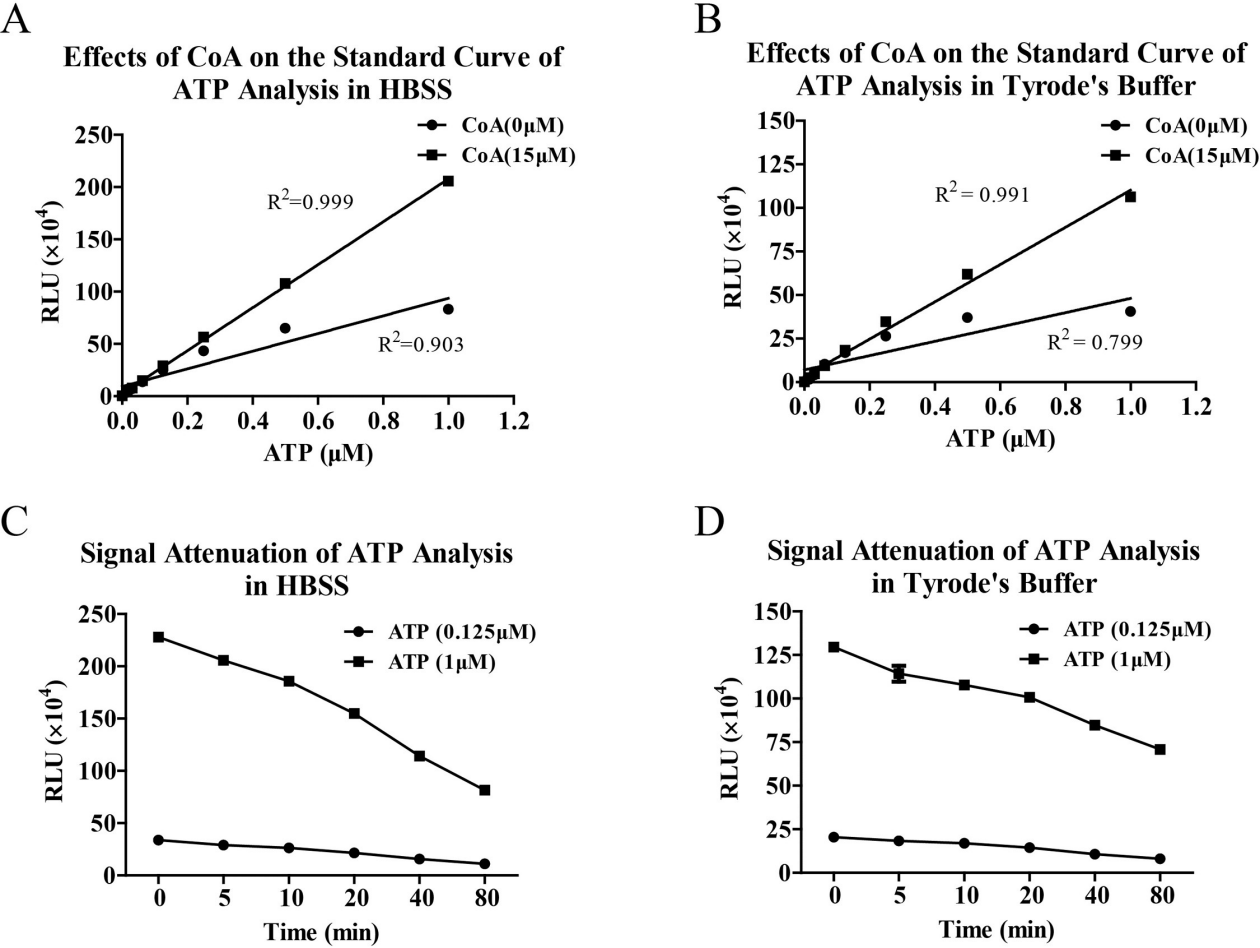
Bioluminescence analyses of ATP in HBSS and Tyrode’s buffer have optimized linearity in the presence of CoA. Bioluminescence analysis of ATP was performed in HBSS and Tyrode’s buffer with or without CoA (15 μM) and standard curves were made (A and B). The signal attenuations of ATP analyses in the presence of CoA (C and D) were also estimated by determining RLU at different time points. Data are representative of three independent experiments.

Although luciferase can maintain high activity in buffer A, platelets cannot keep normal function to be activated by various stimulants, such as thrombin, collagen and ADP. So we further evaluated bioluminescence analyses of ATP in HBSS and Tyrode’s buffer. Consistent with the measurements in buffer A, standard curve linearity of ATP analyses in HBSS and Tyrode’s buffer was significantly improved by adding CoA in the bioluminescence reaction system (Fig 2A and 2B). However, signal attenuations of ATP analyses in HBSS and Tyrode’s buffer were still fast even though they were slower than that in buffer A. The attenuation rates reached approximately 30% in 20 min and 50% in 80 min (Fig 2C and 2D).

### Effects of BSA on the bioluminescence analyses of ATP in HBSS and Tyrode’s buffer

**Fig 3:**
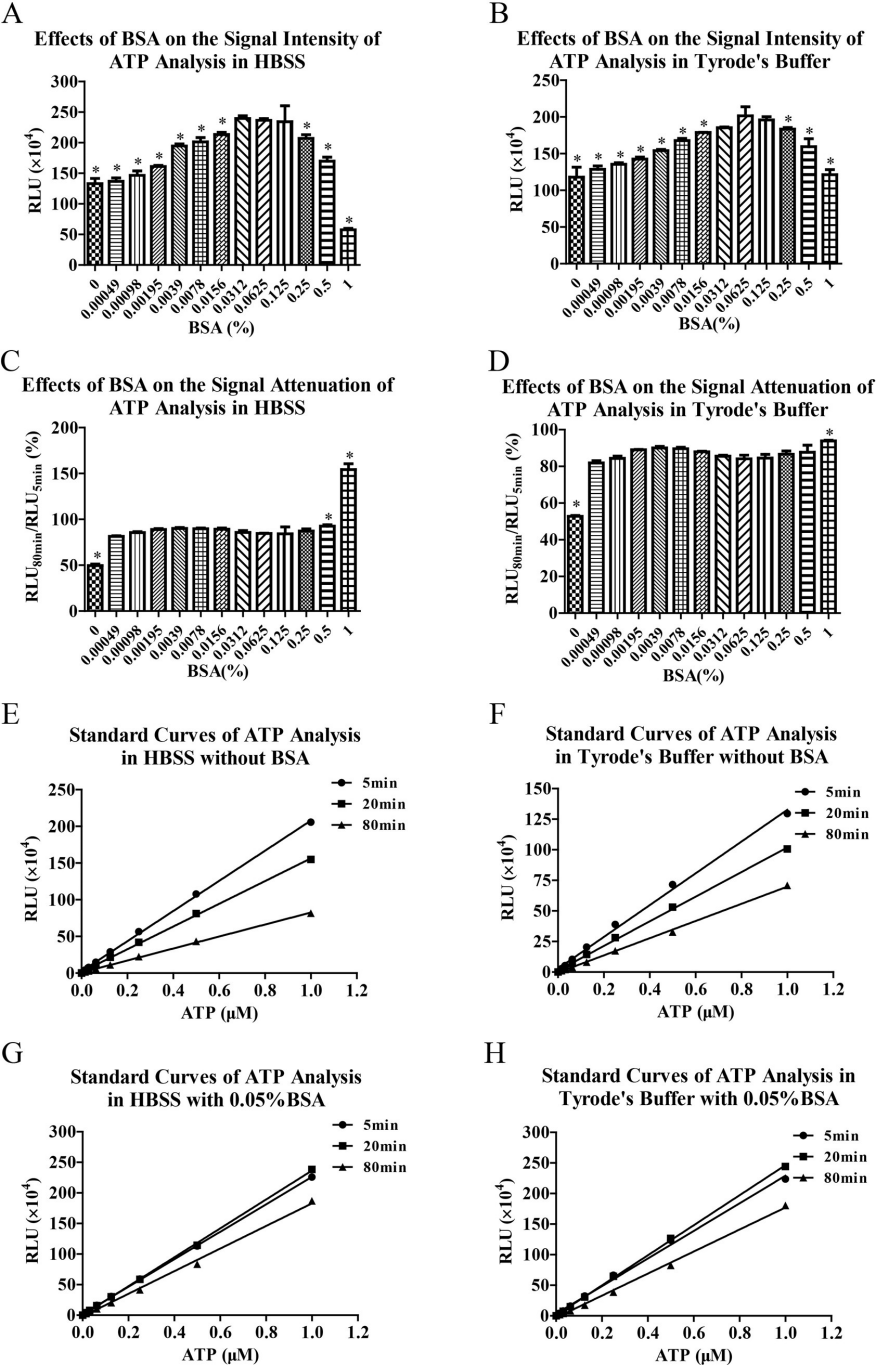
BSA slowed down signal attenuations of bioluminescence analyses of ATP in HBSS and Tyrode’s buffer. Bioluminescence analyses of ATP (1 μM) were performed in HBSS and Tyrode’s buffer in the presence of CoA (15 μM) and serial concentrations of BSA (A and B). The signal attenuations of ATP analyses in the presence of serial concentrations of BSA were also estimated by RLU_80min_/RLU_5min_ (C and D). Then in the presence or absence of BSA (0.05%), standard curve analyses of ATP in HBSS (E and G) and Tyrode’s buffer (F and H) were performed at different time points. Data are representative of three independent experiments. * *P* < 0.05 *vs* BSA (0.0625%).

In view of quickly signal attenuations of ATP analyses in HBSS and Tyrode’s, we always tried to slow down signal attenuation for more accurate ATP analysis. In some experiments, we found that signal attenuation of ATP analysis was retarded by adding 0.35% BSA in the Tyrode’s buffer. So we investigated the effects of BSA on the signal attenuations of ATP analyses in HBSS and Tyrode’s buffer. The results showed that BSA not only enhanced RLU values (Fig 3A and 3B), but also slowed down signal attenuations of bioluminescence analyses of ATP in HBSS and Tyrode’s buffer (Fig 3C and 3D). When BSA was at the concentrations of 0.0312% to 0.125%, the RLU values of ATP analyses were enhanced to the maximum extent (Fig 3A and 3B). As for signal attenuation, BSA reduced signal attenuations at all concentrations, especially at the concentrations of 1% (Fig 3C and 3D). Thereafter, we determined standard curves of bioluminescence analyses of ATP in the presence or absence of 0.05% BSA at different time points. The results showed that signal attenuations of ATP analyses were improved by adding BSA both in HBSS (Fig 3E and 3G) and Tyrode’s buffer (Fig 3F and 3H).

### Effects of BSA on chemiluminescence analysis of HRP and effects of OVA on the bioluminescence analysis of ATP

**Fig 4:**
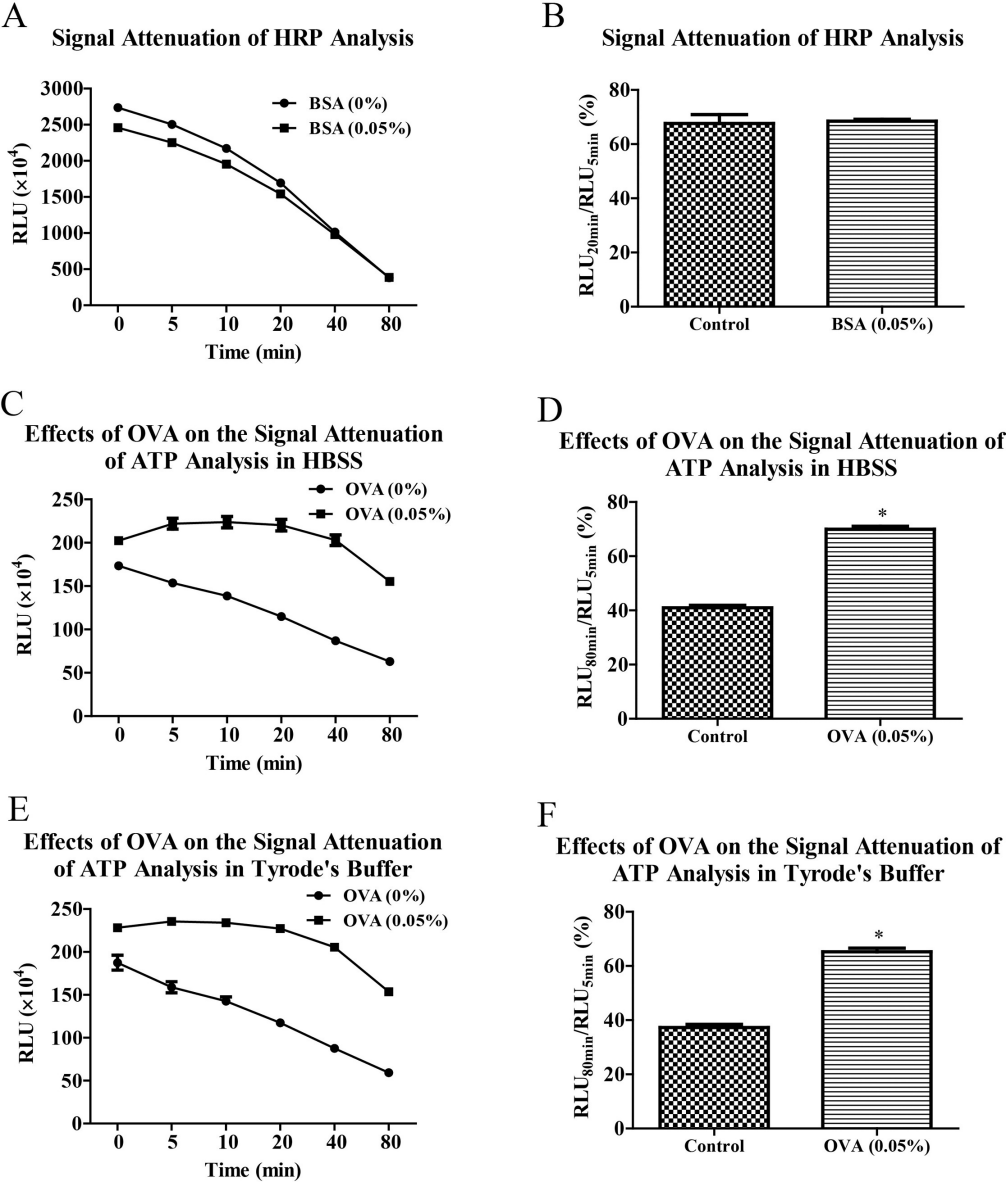
BSA didn’t improve signal attenuation of chemiluminescence analysis of HRP and OVA slowed down signal attenuation of bioluminescence analysis of ATP. In the presence or absence of BSA (0.05%), chemiluminescence analysis of HRP activity (0.001 U/ml) was performed in enhanced chemiluminescence solution at different time points (A) and signal attenuation was evaluated by RLU_20min_/RLU_5min_ (B). Bioluminescence analyses of ATP (1 μM) were carried out in HBSS and Tyrode’s buffer with or without OVA (0.05%) at different time points (C and E). The signal attenuations of ATP analyses were analyzed by RLU_80min_/RLU_5min_ (D and F). Data are representative of three independent experiments. * *P* < 0.05 *vs* BSA (0.05%) or OVA (0.05%).

In order to identify whether the postponed effects of BSA on signal attenuation was specific to bioluminescence analysis of ATP, we followed to evaluate the effects of BSA on chemiluminescence analysis of HRP activity. As shown in Fig 4A and 4B, signal attenuation of HRP analysis hadn’t been slowed down by adding 0.05% BSA in the reaction system and even the signal intensity of chemiluminescence was decreased by BSA. Furthermore, we also investigated the effects of OVA on the signal attenuation of bioluminescence analysis of ATP. Consistent to the effects of BSA, OVA significantly enhanced RLU values and slowed down signal attenuations of ATP analyses in HBSS (Fig 4C and 4D) and Tyrode’s buffer (Fig 4E and 4F).

### Method validation of ATP analysis in the presence of platelets and bioluminescence analysis of ATP released by activated platelets

**Fig 5:**
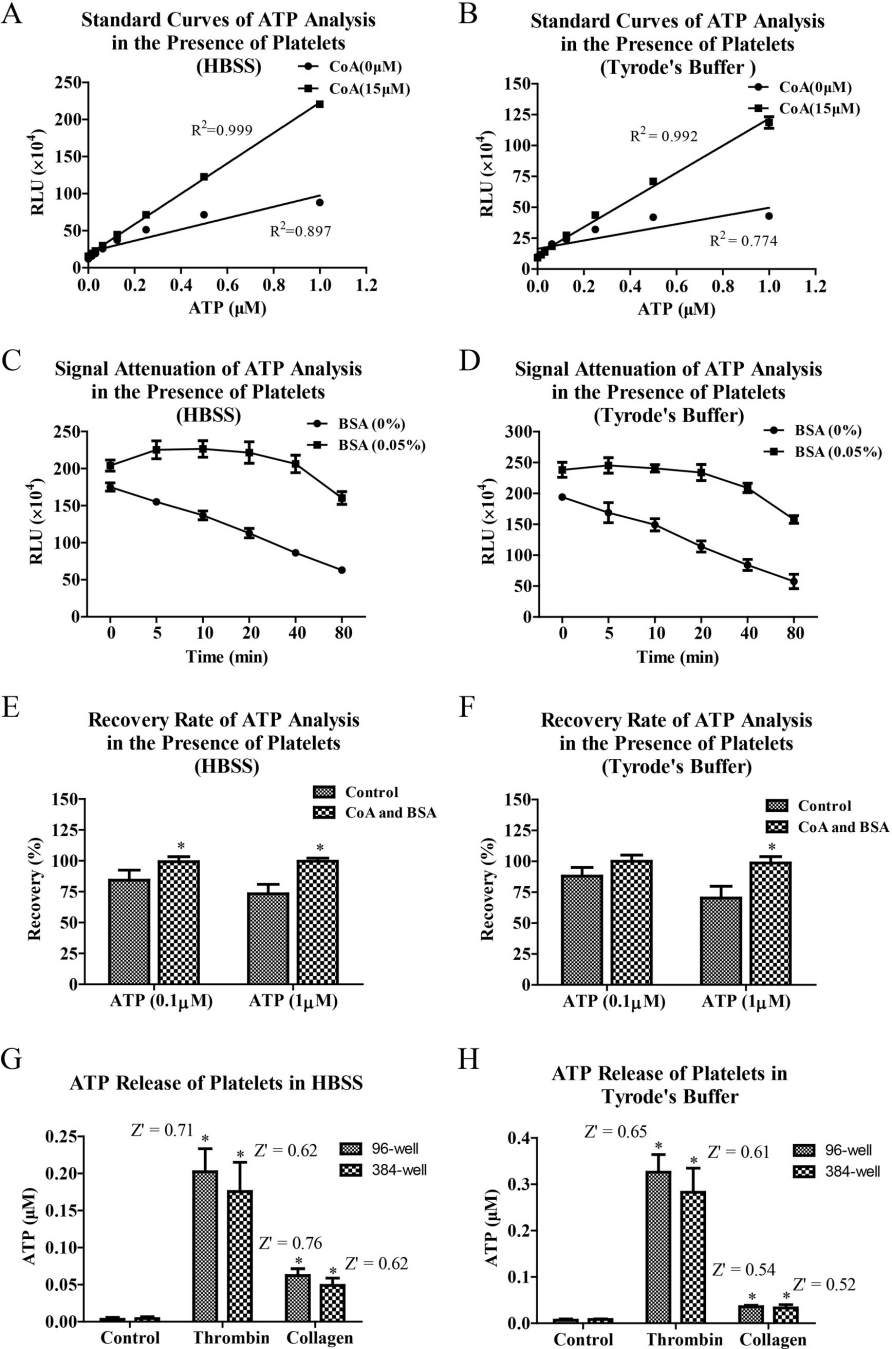
Method validation of ATP analysis in the presence of platelets and bioluminescence analysis of ATP released by activated platelets. Washed platelets were suspended in HBSS or Tyrode’s buffer and seeded in white 96-well plate. In the presence of platelets, standard curves of ATP were performed by adding of CoA (15 μM) into reaction system (A and B) and signal attenuation curves were analyzed by adding of BSA (0.05%) into reaction system (C and D). Data are representative of three independent experiments. Recovery rates of ATP analyses in HBSS and Tyrode’s buffer were analyzed by adding standard ATP (0.1μM or 1μM) into reaction system (E and F). Data are from three independent experiments. At last, the platelets were added into 96-well plate or 384-well plate, and then stimulated with thrombin or collagen at 37°C for 20 min. Bioluminescence analysis of ATP released by activated platelets was initiated by adding luciferase-luciferin work solution containing CoA and BSA at optimized concentrations into 96-well plate and RLU values were determined at 5 min after the start of the reaction (G and H). Concentrations of released ATP and Z’ factors were calculated. Data are from five independent experiments. * *P* < 0.05 *vs* Control.

After bioluminescence analysis of ATP was optimized by adding CoA and BSA in the reaction system, we subsequently validated their optimized effects in the presence of platelets. The results showed that quantitative linearity of ATP analysis was significantly improved through addition of CoA (Fig 5A and 5B) and signal attenuation of bioluminescence was intensively slowed down through addition of BSA (Fig 5C and 5D). In the presence of CoA and BSA, recovery rates of ATP analyses in HBSS and Tyrode’s buffer were also optimized (95% - 105%) compared to control (65% - 90%) (Fig 5E and 5F). Subsequently, the optimized bioluminescence analysis of ATP was used to monitor ATP release of activated platelets. The results (Fig 5G and 5H) showed that ATP releases of activated platelets induced by thrombin and collagen could be well monitored by the optimized bioluminescence analysis of ATP. The ATP releases of platelets in HBSS and Tyrode’s buffer exhibited similar magnitudes. Z’ factors of ATP releases induced by thrombin and collagen in HBSS and Tyrode’s were all over 0.5. Similar results were acquired in experiments of 96-well plate and 384-well plate.

### Screening of platelet inhibitors with optimized bioluminescence analysis of released ATP

**Fig 6:**
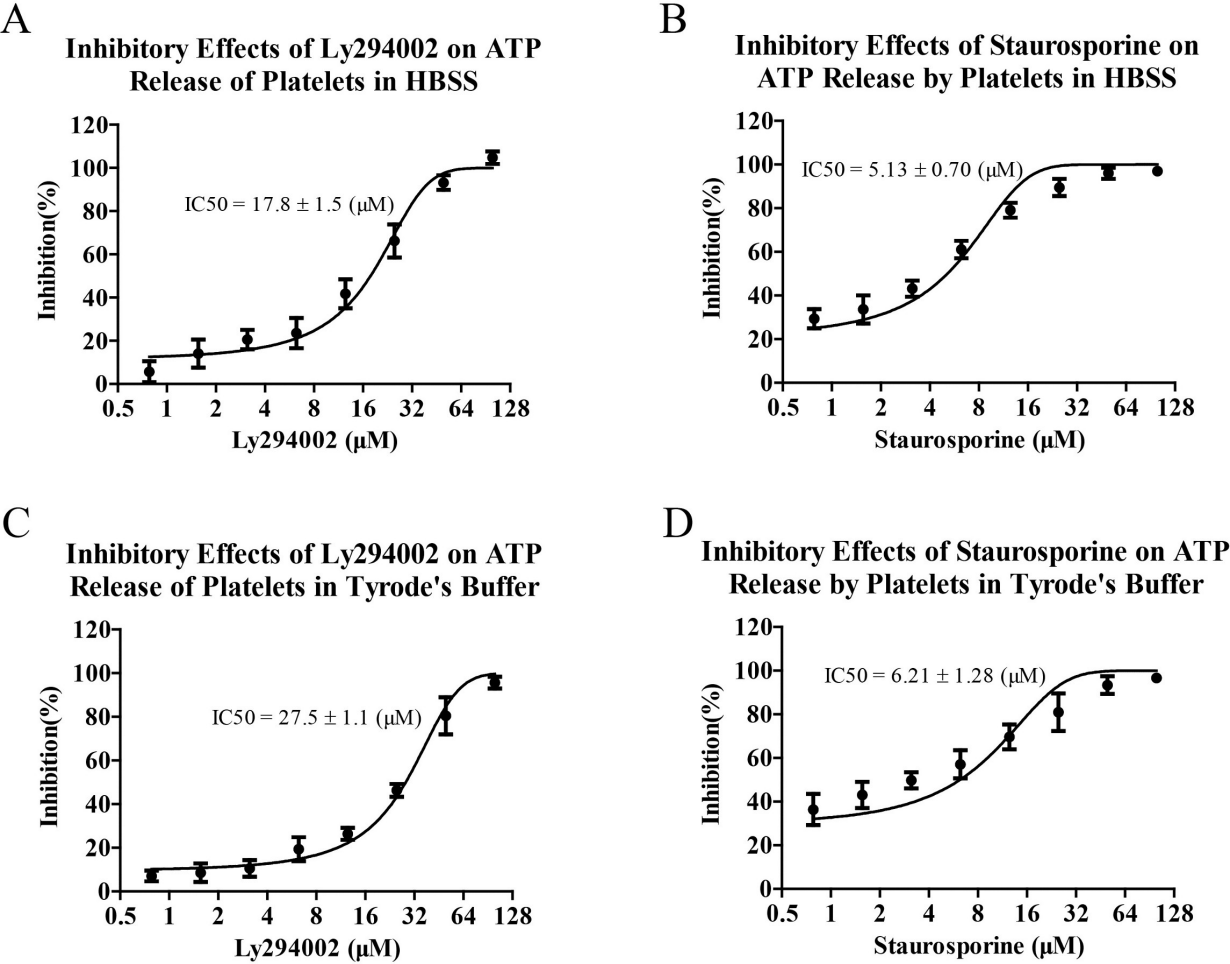
Screening of platelet inhibitors with optimized bioluminescence analysis of released ATP. Washed platelets resuspended in HBSS or Tyrode’s buffer were seeded in white 96-well plate. Subsequently, the platelets were pretreated with Ly294002 or Staurosporine for 5 min and then stimulated with thrombin at 37°C for 20 min. Bioluminescence analyses of ATP released by activated platelets were performed. Inhibition rates and IC50 of Ly294002 (A and B) or Staurosporine (C and D) on ATP release of activated platelets were calculated. Data are representative of three independent experiments.

On the basis of optimized bioluminescence analysis of released ATP, we subsequently analyzed the inhibitory effects of Ly294002 and Staurosporine on the ATP releases of activated platelets and evaluated the application values of bioluminescence analysis of ATP in screening of platelet inhibitors. As shown in Fig 6, both Ly294002 and Staurosporine exhibited inhibitory effects on ATP releases of activated platelets induced by thrombin and they showed similar inhibitory effects in HBSS and Tyrode’s buffer. In addition, it should be noted that the maximum concentration of DMSO in the assay was 0.5% and the effects of DMSO on the platelet analysis was negligible (S1 Fig).

## Discussion

In 1958, CoA was reported to be able to enhance bioluminescence catalyzed by luciferase and the underlying mechanism was considered to be that CoA could remove inhibitory product from enzyme surface and strengthen enzyme reaction [11]. Afterwards, the inhibitory side product in bioluminescence reaction catalyzed by luciferase was identified to be L-AMP and CoA was revealed to induce stabilization of light emission due to the thiolytic reaction between CoA and L-AMP, which give rise to dehydroluciferyl-CoA (L-CoA), a much less power luciferase inhibitor (IC50 = 5 μM) compared with L-AMP [12–14]. Thus, some commercial luciferase reporter assay kits have used CoA to enhance bioluminescence and stabilize light emission in quantitative analysis of luciferase. However, CoA hasn’t been used in bioluminescence analysis of ATP.

In the present study, we firstly performed standard curve assay of ATP in buffer A which contained tricine buffer of pH 7.8 and high concentration meganesium ions, and endowed luciferase suitable catalytic reaction environment. The linear regression analysis showed that standard curve of ATP assay had unsatisfactory linearity. This bad linearity was mainly resulted from low bioluminescence signals when ATP was at high concentrations. After probable effects of luciferase deficiency were eliminated, we proposed that this bad linearity of ATP analysis was originated by inhibitory effects of L-AMP on enzyme reaction. Thereafter, we investigated the effects of CoA on the bioluminescence analysis of ATP and significantly improved standard curve linearity by adding optimized concentration of CoA in the reaction system. However, signal attenuation of ATP analysis was still very fast even in the presence of CoA. This was because signal attenuation of ATP analysis was mainly resulted from consumption of ATP in the catalyzed reaction but not inhibitory effects of L-AMP on enzyme activity.

Generally, maintenance of platelet activity needs suitable buffer and Tyrode’s buffer is often used to determine activation, release, and aggregation of platelets [1–3]. Thus, we furtherly evaluated bioluminescence analysis of ATP in Tyrode’s buffer and HBSS which also possessed glucose and calcium for platelet activation and magnesium for bioluminescence reaction. The results showed that bioluminescence signals of ATP analyses in HBSS and Tyrode’s buffer were lower than that in buffer A, which suggesting that HBSS or Tyrode’s buffer doesn’t provide best reaction conditions for luciferase. The reasons may be relatively low pH values and magnesium ion concentrations in HBSS and Tyrode’s buffer. However, adding CoA in reaction system also significantly improved linearity of ATP analysis in HBSS and Tyrode’s buffer which is consistent with assay in buffer A. So HBSS and Tyrode’s buffer can be used to determine ATP release of platelets by bioluminescence analysis. Of course, there is still the problem of bioluminescence signal attenuation in HBSS and Tyrode’s buffer.

In some experiments, we occasionally found that signal attenuation of ATP analysis was retarded by adding 0.35% BSA in the Tyrode’s buffer for reducing liquid shear force on platelets. Meanwhile, signal intensity of bioluminescence was enhanced by 0.35% BSA. The underlying mechanisms of enhancing bioluminescence signal and postponing signal attenuation in ATP analysis are not clear. As a macromolecule biochemical reagent, BSA is often used to prevent denaturation of many enzymes and cytokines. In some luciferase reporter kit, BSA also used to hinder adsorption of luciferase on multi-well plate and thus keep activity of luciferase. However, we proposed that BSA could play much more meaningful roles in bioluminescence analysis of ATP by slowing down signal attenuation and improving accuracy. Through concentration optimization analysis, we furtherly revealed that mitigation effects of BSA on signal attenuation of bioluminescence were proportional to its concentration and the biggest enhancement effects of BSA on bioluminescence signal were achieved by 0.0312% to 0.125% BSA in HBSS and Tyrode’s buffer. So we used 0.05% BSA to slow down signal attenuation of ATP analysis in later experiments. Based on these results, we also propose a hypothesis that BSA can enhance luminous efficiency of bioluminescence reaction catalyzed by luciferase as done by some cationic liposomes [15, 16] and reduce reaction rate simultaneously. Reduction of bioluminescence reaction rate slows down consumption of ATP and corresponding signal attenuation of bioluminescence while signal intensity is maintained and even enhanced by higher luminous efficiency in the presence of BSA.

In order to investigate the specificity that BSA enhances luminous efficiency and slows down signal attenuation in bioluminescence analysis of ATP, we furtherly evaluated the effects of BSA on chemiluminescence reaction in HRP analysis [17] and the effects of OVA on bioluminescence reaction of ATP analysis. Interestingly, BSA does not slow down signal attenuation of chemiluminescence analysis of HRP and even lowered signal intensity. However, OVA enhanced bioluminescence intensity and postponed signal attenuation in bioluminescence analysis of ATP which was consistent with the effects of BSA. So BSA possesses high specificity in enhancing luminous efficiency and slowing down signal attenuation in bioluminescence analysis of ATP. This specificity is not resulted from preventing denaturation and inactivation of luciferase as usual, but from enhancing luminous efficiency and slowing down bioluminescence reaction rate simultaneously. Especially, the common enhancing effects of BSA and OVA on enhancing luminous efficiency may be contributed by some common amino acid residues. According to report made by Airth et al., cysteine can probably react with L-CoA to produce more stable L-cysteine and furtherly enhance bioluminescence of ATP analysis [11]. Thus the mitigation effects of BSA and OVA on signal attenuation in bioluminescence analysis of ATP may be resulted from united effects of enhancing luminous efficiency by cysteine residues and slowing down bioluminescence reaction rate by barrier effects of BSA and OVA as biological macromolecules. In subsequent platelet experiments, we used CoA to improve linearity and BSA to slow down signal attenuation simultaneously.

Platelet secretion occurs upon activation by specific agonists including thrombin, collagen, ADP, and thromboxane. Previous studies have fully demonstrated that ATP was released from dense granules after thrombin and collagen induced platelet activation [1–3]. In the present study, we further demonstrated that in the presence of platelets, bioluminescence analysis of ATP was significantly optimized by addition of CoA and BSA. Thus, the recovery rates of ATP analyses in HBSS and Tyrode’s buffer were significantly improved by optimized method. Subsequently, we validated that platelets could be activated to release ATP by thrombin and collagen, and bioluminescence analysis of ATP in the presences of CoA and BSA could accurately determine ATP release. No matter in HBSS or Tyrode’s buffer the assays were performed, the Z’ values of ATP analyses were all over 0.5. These results suggest that the optimized bioluminescence analysis of ATP might be potential to be used to screen platelet inhibitors with high throughput. It should be noted that this assay couldn’t be performed in PRP, because PRP would coagulate after stimulated by thrombin and platelets in PRP intensely aggregate after treated with collagen I, then interfering the analysis of ATP release.

Platelet agonists induce activation and aggregation of platelets and following thrombus formation through stimulating multiple signaling pathways [18–25]. After optimized bioluminescence analysis of ATP and evaluated its availability in ATP release analysis of platelets, we validated its application values in the high throughput screening of platelet inhibitors by analyzing inhibitory effects of PI3K inhibitor and PKC inhibitor on ATP release of platelets. Consistent with expectation, inhibitory effects of PI3K inhibitor and PKC inhibitor on ATP release of platelets induced by thrombin were perfectly determined by optimized bioluminescence analysis of ATP. PI3K inhibitor or PKC inhibitor had similar IC50 values on ATP releases of platelets in HBSS and Tyrode’s buffer.

Collectively, we have successfully optimized bioluminescence analysis of ATP by improving standard curve linearity with CoA and postponing signal attenuation with BSA. This optimized method can be used to accurately determine activation of platelets and screen platelet inhibitors with high throughput in HBSS or Tyrode’s buffer.

## Supporting information

S1 Fig The effects of DMSO on ATP release of platelets. Washed platelets were suspended in Tyrode’s buffer and seeded in white 96-well plate. Different percentages of DMSO were added into platelet suspension for 20 min. Bioluminescence analysis of ATP released by platelets was initiated by adding luciferase-luciferin work solution containing CoA and BSA at optimized concentrations into 96-well plate and RLU values were determined at 5 min after the start of the reaction. Data are from three independent experiments. * *P* < 0.05 *vs* Control.
(TIF)
