# Supplementary figures and images for: Optimized bioluminescence analysis of adenosine triphosphate (ATP) released by platelets and its application in the high throughput screening of platelet inhibitors
Source: PLoS One. 2019 Oct 10;14(10):e0223096. doi: 10.1371/journal.pone.0223096 (PMC6786574; doi:10.1371/journal.pone.0223096)

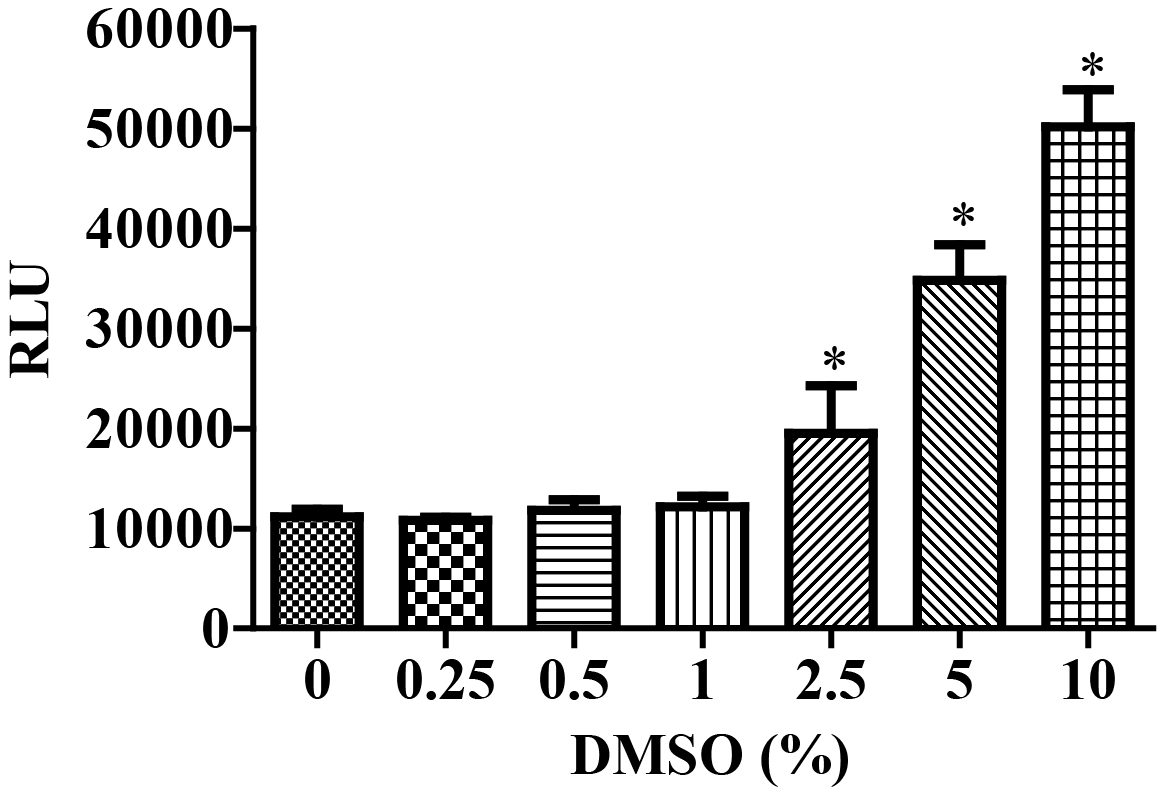

Supplement: S1 Fig — Washed platelets were suspended in Tyrode’s buffer and seeded in white 96-well plate. Different percentages of DMSO were added into platelet suspension for 20 min. Bioluminescence analysis of ATP released by platelets was initiated by adding luciferase-luciferin work solution containing CoA and BSA at optimized concentrations into 96-well plate and RLU values were determined at 5 min after the start of the reaction. Data are from three independent experiments. * P < 0.05 vs Control. (TIF) [file pone.0223096.s001.tif]
